# The effect of open-label placebo on anxiety, cortisol, and pain levels in children with procedural pain

**DOI:** 10.3389/fped.2026.1720751

**Published:** 2026-03-27

**Authors:** Anna Lena Friedel, Tim Szallies, Jana Oberlohmann, Leonie Peters, Sven Benson, Manfred Schedlowski, Eva Tschiedel

**Affiliations:** 1 Institute of Medical Psychology and Behavioral Immunobiology, Center for Translational Neuro- and Behavioral Sciences (C-TNBS), University Hospital Essen, University of Duisburg-Essen, Essen, Germany; 2 Department of Health, Social Affairs & Education, CBS University of Applied Science, Cologne, Germany; 3 Department of Pediatrics I, University Hospital Essen, University of Duisburg-Essen, Essen, Germany; 4 Institute for Medical Education, C-TNBS, University Hospital Essen, University of Duisburg-Essen, Essen, Germany; 5 Department of Clinical Neuroscience, Osher Center for Integrative Medicine, Karolinska Institutet, Stockholm, Sweden

**Keywords:** anxiety, pain, pediatrics, placebo, stress, open-label placebo

## Abstract

**Purpose:**

Needle-related procedures are painful experiences for hospitalized children and are associated with anxiety, pain and stress. Open-label placebos (i.e., placebos administered honestly as an inert substance) have recently been shown to be as effective as deceptive placebos in inducing placebo analgesia. This study systematically investigated the impact of an open-label placebo intervention on anxiety, pain and stress during venipuncture in children.

**Patients and methods:**

Forty-seven children and adolescents aged 8–14 years participated in this randomized controlled trial. Prior to venipuncture, patients in the intervention group watched a video introducing the placebo juice, and drank it. The control group received standard care. Saliva cortisol levels as well as anxiety and pain were assessed using validated questionnaires before the venipuncture, shortly afterwards and one hour later.

**Results:**

The placebo intervention did not result in significant changes in pain perception, anxiety or saliva cortisol levels. However, we observed positive correlations between anxiety and pain, as well as between baseline cortisol levels and post-venipuncture anxiety and pain.

**Conclusion:**

In this pilot-study setting, an open-label placebo intervention did not reduce pain, anxiety or saliva cortisol concentrations. The discrepancy between these initial pilot data and those of experimental studies suggests that numerous real-world factors may influence the placebo effect. Further research should identify these factors to develop strategies that maximize the therapeutic potential of the placebo effect for this patient population in clinical practice.

**Clinical Trial Registration:** https://drks.de/register/de/trial/DRKS00029938/preview, identifier (DRKS-ID: DRKS00029938).

## Introduction

The placebo effect, defined as a positive health outcome following the administration of an inert treatment, has been scientifically acknowledged since 1955 (1, 2). Importantly, placebo effects also occur in effective drug treatment and can increase treatment efficacy (2). Placebo analgesia in adults is well-documented and has been integrated into clinical guidelines for perioperative and posttraumatic pain management in Germany since 2015 (3). Its mechanisms are multifactorial, involving expectations, conditioning, learning, memory, motivation, somatic focus, reward, anxiety reduction and meaning (2). Expectation in particular has a huge influence on the effect of placebo administration (4, 5). It is assumed that an additional element of the analgesic placebo effect involves a reduction in anxiety through changes in expectation (6). Nevertheless, some aspects of placebo analgesia still require clarification.

Recently, initial studies have suggested that deception is not necessary to elicit a placebo response. In these studies, placebos (i.e., inert substances such as placebo pills) have been administered openly, and patients are honestly informed that they will receive an inert substance that activates bodily processes that can alleviate symptoms like pain (7, 8). Very little is known about open label placebo analgesia in children. Only in recent years has Kaptchuk's working group conducted studies on this topic. The results so far show that open label placebo is also effective in children and adolescents (9). Another study by the working group on this topic is currently underway, but no results have been published yet (10).

In general, possible positive effects of placebo analgesia were mainly shown in experimental settings. A review of the limited available literature revealed that the placebo effect in children generally might exceed that in adults whilst the underlying mechanisms need to be elucidated using pediatric-specific methods (11).

One experimental study of healthy schoolchildren could show that applying a placebo cream suggested to relieve pain, significantly increased both the heat pain threshold and tolerance. This effect was 3.6–5.6 fold higher than that observed in adults (12). A second experimental study, conducted under similar conditions, examined the differences in the placebo analgesic effect between children and adults. This study confirmed the efficacy of placebo analgesia in children, though it did not demonstrate a difference in extent compared to adults (13). The most recent experimental study in this field, consistent with the previous two studies, showed that the topical application of placebo cream induced analgesia in response to phasic heat stimuli in healthy pediatric study participants. In this study, the effect of placebo hypoalgesic effect was greatest in children younger than 14 years old compared to adolescents and adults (14).

One single pediatric clinical study compared the analgesic effects of either topical application of placebo cream, topical application of placebo cream plus verbal suggestion, and no cream during venipuncture. No clear effect was observed in this study (15).

Needle procedures are common in everyday clinical practice. Various methods, including the use of topical or systemic analgesics, as well as physical (e.g., positioning, sucking) and psychologic (e.g., preparation, distraction) interventions are regularly used to reduce needle pain and have been proven helpful (16–20). Nevertheless, pain remains a prevalent topic in pediatric clinics. During hospital stays, 37%–53% of children report having untreated pain (21–23) with needle-procedures reported to be worst (16, 21). In addition to pain, anxiety and stress are pervasive for children in hospital settings. These emotional states are interdependent and difficult to separate (24, 25). Since anxiety increases pain perception, influencing anxiety level may offer an important approach for influencing pain (26). Additionally, high stress levels increase pain perception, and conversely, pain increases stress levels (27).

A well-established biomarker to quantify stress levels is cortisol, a hormone produced by the adrenal gland in response to various stressors (28, 29). Since saliva cortisol levels are proportional to serum cortisol levels, saliva cortisol is an especially suitable stress marker in children because it is non-invasive and easy to collect (30). Pain perception and increased cortisol levels influence each other. On the one hand, the presence of pain leads to an increased release of cortisol (28), but on the other hand, elevated cortisol levels also increase the sensitivity to pain (31).

State anxiety (i.e., acute situational anxiety) can be modulated by various factors such as environmental aspects (e.g., colors, room design, music), communication and caregiver's behavior (27, 32, 33). Perceiving the environment as unsafe, feeling inferior to adults, fearing loss of control, and experiencing shame and surrender are core aspects of children's discomfort (34). The positive processing of these frightening situations is crucial for children as it can have an impact on their future behavior in similar settings, their physical development and their individual pain threshold (35–37).

Overall, stress, anxiety and pain mutually reinforce each other. This combination can lead to considerable discomfort and significantly impact on the physical and psychological development of children. All three entities are treatable with placebo, as evidenced in previous research (38). For the first time, this study aims to test the efficacy of open-label placebo in an everyday setting on a pediatric ward. By choosing a placebo juice as part of an open label placebo design, we deliberately opted for a different method other than topical application which is easier to use in everyday clinical practice: Since puncture sites and puncture times are often uncertain conditions the use of placebo-juice is beneficial. It can be applied *ad hoc* and does not require an hour of application time (as is the case with EMLA). Additionally, in general, open label placebo offers a significantly wider range of application options than a cream.

We specifically hypothesized that the placebo intervention would reduce saliva cortisol concentrations as an established indicator of acute stress, as well as self-reported state anxiety and pain during venipuncture. In addition, associations between cortisol, anxiety, and pain were assessed in exploratory analyses as secondary outcomes.

## Patients and methods

### Study participants

The present study is a monocentric, prospective, randomized controlled trial. Between August 2022 and October 2023, 47 children and adolescents with a median age of 11 years (9; 13) (range 8–14 years) were included. Patients were eligible for recruitment when their admission was elective and the primary treating department provided indication for venipuncture. Inclusion criteria were age between eight and 14 years and age appropriate development. Exclusion criteria were: unable to provide information, chronical pain, insufficient German language skills, receiving corticosteroid-containing medication, receiving anxiolytics or analgesics the same day. The study was prospectively registered before inclusion of the first patient in the German Clinical Trials Registry (https://drks.de/search/de/trial/DRKS00029938/details; registration date 08/02/22) and approved by the local ethics committee (vote 22-10517-BO; 06/15/22). Please note that the originally targeted sample size of *N* = 140 participants could not be achieved due to strict exclusion criteria and a low approval rate from parents or legal guardians. A *post-hoc* power analysis for the analyzed sample size yielded a power of approximately 0.8 (1–*β* = 0.78), with *α* < .05 (one-tailed) for detecting a large effect size (d = .80). Therefore, it can be assumed that the current sample size was sufficient to detect at least large and clinically relevant effects, while smaller or more subtle placebo effects cannot be ruled out.

### Study design

After the parents or legal guardians of the participants signed written informed consent form, participants were randomly assigned to intervention or control group. Children in the control group received standard care and were told that the aim of the study was to find out how comfortable and relaxed they felt during the venipuncture and for this purpose were asked to complete questionnaires and provide saliva samples. They were not informed that they were in the control group and would not receive an additional placebo treatment in order to avoid disappointment and its influence on the results. In the intervention group, positive treatment expectations were to be induced by combining elements of open-label placebo administration and social observational learning (see below for details).

**Figure 1:**
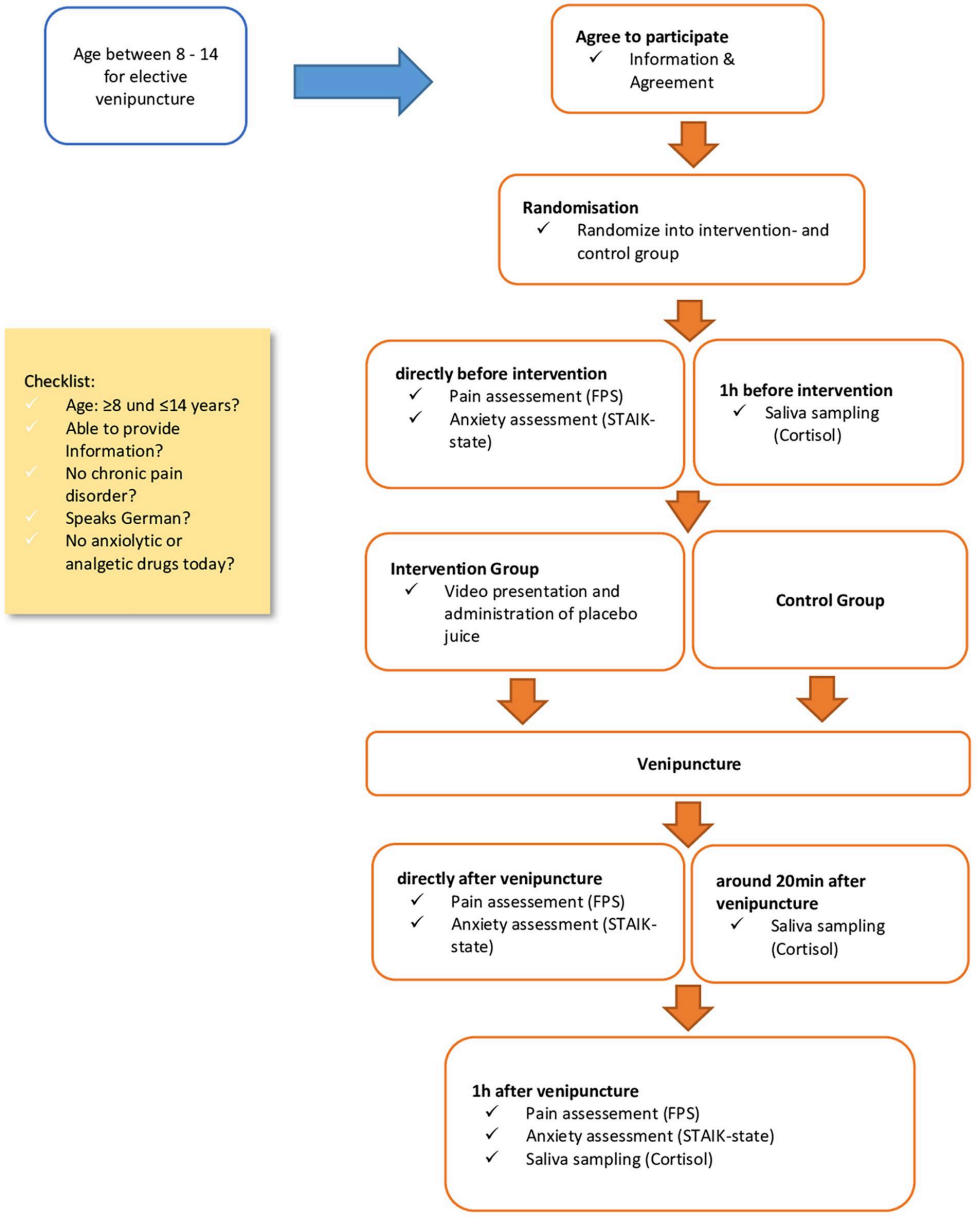
Study flowchart.

In both groups, anxiety and pain were measured directly before venipuncture (t0), directly after (t1), and approximately one hour after (t2) venipuncture. Saliva sample for the assessment of cortisol concentrations were collected one hour before venipuncture (t0) as well as around 20 min (t1) and one hour (t2) after the venipuncture (Figure 1).

### Placebo intervention

Immediately before the venipuncture, the children in the intervention group were given a purple juice. Based on other studies [e.g., (9)], it was openly explained that this juice was a placebo containing no active ingredients. To further induce positive expectations, the children were given the verbal information that the placebo effect has been scientifically proven to be helpful in many areas, and can therefore have a positive effect on medical treatment, leading to a reaction in the body similar to that caused by medication, which is is, so to speak, a superpower of the placebo juice. The children also watched a 2:30 min video combining cartoon elements with footage of two children acting as if they had been given the “magic juice”, which made them feel very comfortable and relaxed during a medical procedure. The video's approach was based on findings regarding social observational learning of positive expectations, which have been shown to have positive effects on adults experiencing pain (39), and which could “boost” the placebo effect (40).

### Behavioral measures

To quantify pain intensity, a combination of a numeric rating scale (NRS) and a faces pain scale (FPS) was employed. These have been empirically documented to be well-qualified for the subjective assessment of pain in children (41, 42). The NRS ranges from 0 (“I feel very well, I have no pain at all”) to 10 (“I feel very badly, I have very severe pain”) with a total of 6 additional facial expressions for the steps 0, 2, 4, 6, 8, 10.

One validated instrument to measure anxiety in children is the State Trait Anxiety Inventory for Children (STAIK). The STAIK is a questionnaire that assesses both a general proneness to anxious behavior rooted in the personality of the child (trait anxiety, STAIK-T) and anxiety as a fleeting emotional state induced by a certain situation (state anxiety, STAIK-S) (43). Both scales consist of 20 items, each of which has to be rated on a 3-point Likert scale. The sum score of each scale is between 20 and 60.

### Cortisol

Saliva for cortisol analysis was collected with commercial collection devices (Salivette Cortisol; Sarstedt, Nümbrecht, Germany) and stored at −20 °C until analysis. Saliva cortisol levels were measured by enzyme-linked immunosorbent assay (Cortisol Saliva ELISA, IBL International, Hamburg, Germany) according to the manufacturer's instructions. Cross-reactivity of the anti-cortisol antibody with other relevant steroids was 7% (11-desoxycortisol), 4.2% (cortisone), 1.4% (corticosterone), 0.4% (progesterone), and <0.01% (testosterone). Sensitivity of the assay was 0.082 nmol/L and mean inter- and intra-assay CVs were <10%.

### Statistical analysis

Data were analysed using PASW statistics (SPSS, version 29). The Shapiro–Wilk-Test was conducted to determine whether the data met the assumption of normality. Since this criterion was not met for most of the parameters, non-parametric calculations were performed.

Sociodemographic and psychological characteristics of the experimental and control group were compared using Mann–Whitney *U*-test or *χ*^2^-tests, respectively.

To investigate possible differences between the two groups in cortisol concentrations as well as anxiety levels and pain perception at the different measurement time points, the Mann–Whitney *U*-test was used again. Friedman ANOVA was performed to analyze changes over time within the groups. Where significant results were found, the Wilcoxon-test was used *post-hoc* for individual comparisons to baseline level within the groups. Spearman correlation was performed to investigate possible associations between cortisol, anxiety and pain for secondary outcomes.

Bonferroni correction was applied for multiple comparisons. The level of significance was set at *p* < .05 (for multiple comparisons *p* < .05/number of comparisons, respectively). In addition, effect sizes are reported as r (for Mann–Whitney-*U* and Wilcoxon-test) and Cramer's V (for *χ*^2^-test). Data are given as median (25th; 75th percentiles), for reasons of clarity, in the graphs data are represented by mean ± standard deviation (SD).

## Results

A total of *N* = 47 participants were recruited for this study. They had a median age of 11 years (9; 13) (range 8–14 years). Since the experimental (*n* = 20) and control group (*n* = 27) differed significantly in age (*Z* = −2.78, *p* = .006, *r* = −0.41) and non-parametric calculation methods do not allow for inclusion of age as a covariate, the sample was age-matched to eliminate age as a confounding variable. To this end, participants in each group were sorted in a descending order of age and time of participation in each group, and the seven oldest participants were excluded. The seven eliminated participants had the following characteristics: Four were female, three were male, six of them were 14 years old, one was 13 years old. The elimination resulted in two equally sized groups (both *n* = 20) with a median age of 11 years (9; 12) (range 8–14 years), that did not differ significantly in age (Z = −1.69, *p* = .092, r = −0.27). Sex distribution was equal in the matched groups: 50.0% of the age-matched sample (*n* = 20) were female, 50.0% (*n* = 20) were male [*χ*^2^(1) = 1.60, *p* = .206, V = .20]. Further, intervention and control group did not differ in trait anxiety before the venipuncture (Z*_t0_* = −0.89, p*_t0_* = .375, r*_t0_* = −0.14). They did not differ in state anxiety (Z*_t0_* = −0.56, p*_t0_* = .592, r*_t0_* = −0.09), current pain perception (Z*_t0_* = −0.40, p*_t0_* = .689, r*_t0_* = −0.06) or cortisol level (Z*_t0_* = −0.44, p*_t0_* = .663, r*_t0_* = −0.07) before the venipuncture (see Table 1).

**Table 1:** Sociodemographic, psychological and biological characteristics of the experimental and control group at baseline.

Characteristic parameters	Experimental group (*n* = 20)	Control group (*n* = 19–20)	*p*-value
sex (% female/male)	60.00/40.00	40.00/60.00	.206
age	9.50 (9.00; 11.75)	11.00 (9.00; 12.75)	.092
STAIK trait anxiety	35.00 (29.25; 40.25)	34.00 (27.00; 40.00)	.375
STAIK state anxiety	32.50 (31.00; 45.75)	32.00 (28.00; 44.00)	.592
FPS pain perception	0.0 (0.0; 0.0)	0.0 (0.0; 0.0)	.689
Salivary cortisol (µg/dL)	0.13 (0.07; 0.23)	0.11 (0.08; 0.19)	.663

STAIK, State Trait Anxiety Inventory for Children; FPS, Faces Pain Scale. Data are reported as median (25th; 75th percentiles) if not otherwise indicated.

**Figure 2:**
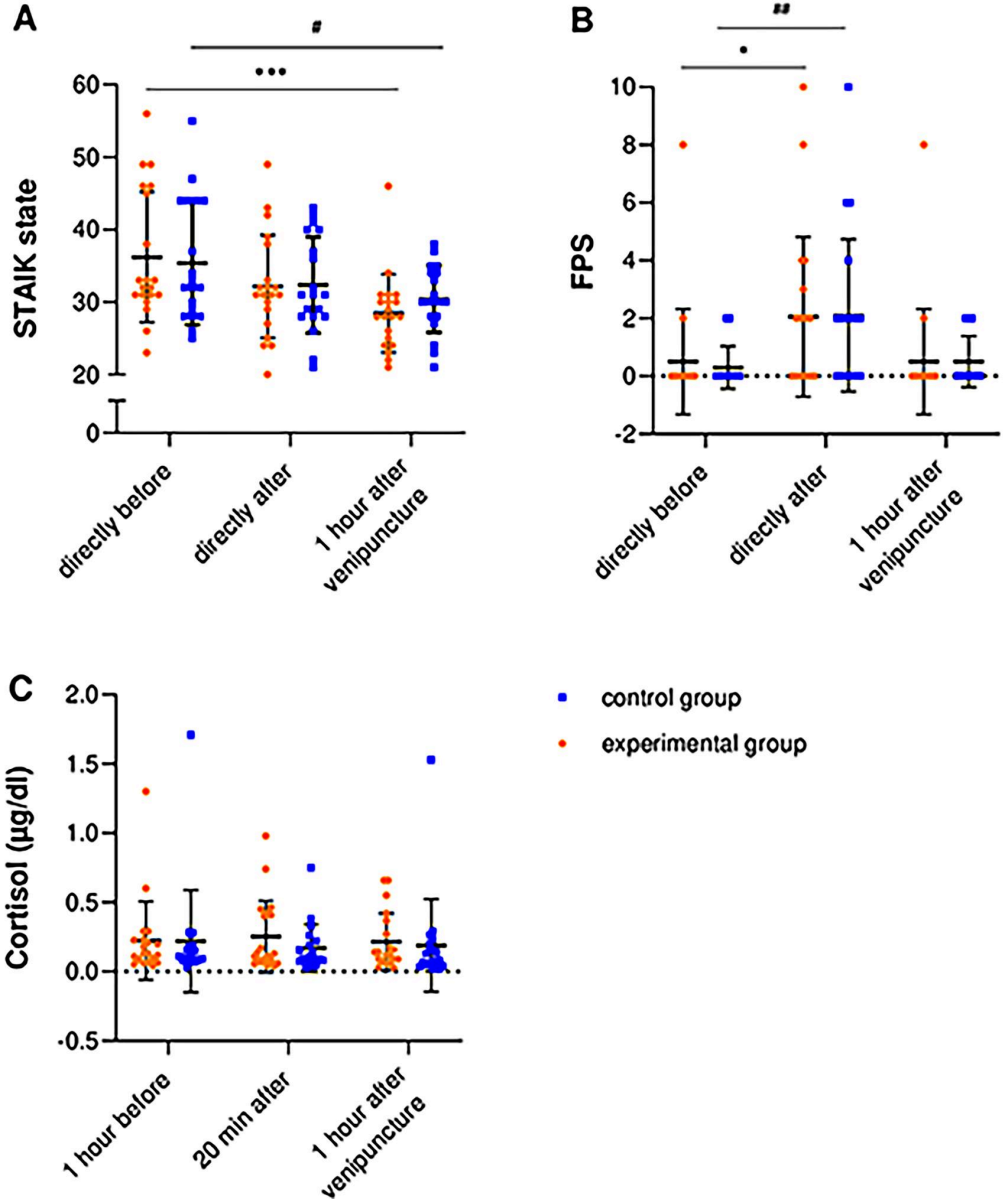
**(A)** State anxiety (STAIK state), **(B)** pain perception (FPS) and **(C)** cortisol levels at baseline, 20 min after and one hour after the intervention. Data are shown as mean ± SD. * *p* < .05, *** *p* < .001 (significant changes within the experimental group), # *p* < .05, ## *p* < .01 (significant changes within the control group). STAIK, State Trait Anxiety Inventory for Children; FPS, Faces Pain Scale.

No significant differences were found between the groups 20 min or one hour after the venipuncture with regard to state anxiety (Z*_t1_* = −0.04, p*_t1_* = .966, r*_t1_* = −0.01; Z*_t2_* = −1.61, p*_t2_* = .107, r*_t2_* = −0.26) or pain perception (Z*_t1_* = −0.13, p*_t1_* = .897, r*_t1_* = −0.02; Z*_t2_* = −1.13, p*_t2_* = 0.260, r*_t2_* = −0.18) (see Figures 2A,B). Similarly, no significant differences in cortisol concentrations were found 20 min or 60 min after the venipuncture (Z*_t1_* = −0.91, *p_t1_* = 0.361, r*_t1_* = −0.15; Z*_t2_* = −1.17*_t2_*, p*_t2_* = 0.243, r*_t2_* = −0.19) (see Figure 2C).

However, significant changes were found within the groups. Participants of the experimental and control group showed a reduction in state anxiety over time. This resulted in significantly lower state anxiety levels one hour after the venipuncture compared to one hour before the venipuncture (experimental group: Z = −3.52, *p* < 0.001, r = −0.79; control group: Z = −2.41, *p* = .016, r = −0.55) (see Figure 2A). Furthermore, both groups demonstrated significantly increased pain perception 20 min after the venipuncture (experimental group: Z*_t1_* = −2.65, p*_t1_* = .008, r*_t1_* = −0.59; control group: Z*_t1_* = −2.91, p*_t1_* = .004, r*_t1_* = −0.65). The pain level decreased over time, approaching the baseline level (Figure 2B). No significant changes in cortisol levels were found within the two groups [experimental group: *χ*^2^(2) = 1.60, *p* = .449; control group: *χ*^2^(2) = 2.84, *p* = .241].

For the sake of completeness, these analyses were also performed on the full sample, without excluding the seven participants who were excluded due to age matching. This had no effect on the reported outcomes (data not shown).

As no differences between the two groups were found for any of the parameters analyzed, for secondary outcomes Spearman correlation was performed for the total group of *n* = 40 participants and revealed an association between state anxiety and pain perception. Directly before the venipuncture, reports of higher pain were associated with higher levels of state anxiety (r_s_ = .402, *p* = .011). A similar association was also observable one hour after the venipuncture (r_s_ = .618, *p* < .001). Furthermore, state anxiety and pain perception were both significantly correlated with cortisol levels. Higher cortisol level one hour before the venipuncture were associated with greater state anxiety (r_s_ = .388, *p* = .016) and higher pain ratings (r_s_ = .386, *p* = .015) assessed immediately before the venipuncture as well as with state anxiety measured directly after the venipuncture (r_s_ = .363, *p* = .025). Furthermore, higher cortisol levels 20 min and one hour after the venipuncture correlated with higher state anxiety levels (20 min: r_s_ = .439, *p* = .006, one hour: r_s_ = .350, *p* = .031) and pain ratings (20 min: r_s_ = .509, *p* < .001; one hour: r_s_ = .472, *p* = .002) assessed one hour after the venipuncture.

## Discussion

This randomized controlled trial aimed to evaluate the efficacy of an open-label placebo intervention in reducing anxiety, stress and procedural pain during venipuncture in children. Groups were carefully matched and did not differ in any sociodemographic, psychological or biological characteristic.

Contrary to our initial hypothesis, however the results did not yield a significant reduction in cortisol concentrations, anxiety or pain in the experimental group compared to the control group. Overall, state anxiety was lower one hour after venipuncture than before venipuncture. In addition, all patients’ pain level initially increased in both groups and then returned to baseline. Exploratory analyses revealed that children in both groups reported greater pain when their state anxiety and cortisol levels were higher. They were also more anxious when their cortisol levels were higher. These findings correspond to the well-known correlation between negative emotions, and especially fear, and an increased perception of pain (44, 45). They are also consistent with the results of numerous studies that evaluated the influence of stress on the perception of pain (31, 46).

The absence of a placebo analgesic effect in the present study may be explained by the limitations of our study: First, we could not provide a standardized and quiet environment for the placebo procedure and venipuncture. Due to organizational limitations at our hospital, children had to change the rooms repeatedly and were seen by different doctors and nurses. They were also exposed to restlessness and loudness in the hospital environment. Unfortunately, these conditions are standard in everyday clinical practice and therefore close to the reality of the conditions under which a potential placebo intervention would be used. But since situations that children perceive as threatening or stressful can be positively influenced by environmental factors (27, 32), the absence of these stabilizing factors may have considerably interfered with the intended placebo effect. Second, expectations and beliefs of study participants were not measured. Therefore, we cannot rule out the possibility of inconsistent manipulation, which limits the interpretation of the null findings. Third, the presence of parents and caregivers during the painful intervention was neither documented nor evaluated. Since it is common for parents to stay with their children during painful procedures at our hospital, it is reasonable to assume that they were present throughout the entire admission and treatment process. However, their influence was neither recorded nor verified pre-, peri- or post-venipuncture. The anxiety reduction resulting from their presence and devotion alone could have masked any potential benefit of the placebo intervention. Fourth, it was also not surveyed if the children had prior experience with painful medical procedures or possible trauma. It is well known that, compared to adults, previous experiences have a particularly significant impact on placebo responses in children (13, 20). Since our university hospital treats many children with chronic diseases and repeated hospitalizations, negative prior experiences and corresponding expectations might have been present and therefore acted as confounding variables. Fifth, the control group did not receive as much attention as the experimental group, which may have already caused a bias.

Various additional factors may have contributed to our findings: For example, Gniss et al. showed that parental instruction, suggestion and administration of placebo medication can significantly amplify the placebo effect in children (14). This effect was not used in our study setting. All explanations and suggestions, as well as the administration of the placebo intervention were performed by medical students. Finally, the actual sample size was sufficient to detect only large effects. Even small differences might have become significant with the originally planned sample, although their clinical significance would then have to be critically evaluated.

All pediatric studies on placebo analgesia aimed to reduce the perception of localized pain induced by a thermal stimulus or needle procedure. For this purpose, a topical placebo cream was used (12–15). Our study was the first to use a systemically applied placebo. Assuming that we did not succeed in reducing anxiety and cortisol concentrations with this intervention due to the aforementioned limitations, it may not be an effective method in reducing puncture pain.

Although the primary outcomes of this pilot-study were not statistically significant, the study provides valuable insights into the complexities of pain management in children. The absence of a significant placebo effect in this context requires further investigation under standardized environmental conditions. Furthermore, the influencing factors for placebo efficacy such as child age, prior experiences with medical procedures, placebo application method and the rationale to use placebo (i.e., the “cover story”) should be systematically considered. This would allow for a more nuanced understanding how open-label placebo interventions could reduce stress, anxiety and pain in children during medical procedures.

In conclusion, these initial pilot-data do not indicate that an open-label placebo intervention reduces cortisol concentrations, anxiety, or procedural pain in children undergoing venipuncture. Since these results differ from results of those studies in experimental settings, it is conceivable that various factors may reduce the well-established efficacy of placebo effects in real life. It is crucial that further translational studies identify these factors in order to develop applicable concepts to make the best possible use of the undoubtedly existing placebo effect for this patient collective in clinical practice.

## Data Availability

The raw data supporting the conclusions of this article will be made available by the authors, without undue reservation.
